# The Relationship between Executive Functions and Language Production in 5–6-Year-Old Children: Insights from Working Memory and Storytelling

**DOI:** 10.3390/bs10020052

**Published:** 2020-02-05

**Authors:** Aleksander Veraksa, Daria Bukhalenkova, Natalia Kartushina, Ekaterina Oshchepkova

**Affiliations:** 1 Lomonosov MSU, Moscow 125009, Russia; veraksa@yandex.ru; 2 Department of Psychology, University of Oslo, 0317 Oslo, Norway; natalia.kartushina@psykologi.uio.no; 3 Institute of linguistics, Russian Academy of Sciences, Moscow 125009, Russia; maposte06@yandex.ru

**Keywords:** preschool age, verbal working memory, visual working memory, oral language, narrative

## Abstract

This study examined the relationship between working memory capacity and narrative abilities in 5–6-year-old children. 269 children were assessed on their visual and verbal working memory and performed in a story retelling and a story creation (based on a single picture and on a series of pictures) tasks. The stories were evaluated on their macrostructure and microstructure. The results revealed a significant relationship between both components (verbal and visual) of working memory and the global indicators of a story’s macrostructure—such as semantic completeness, semantic adequacy, programming and narrative structure—and with the indicators of a story’s microstructure, such as grammatical accuracy and number of syntagmas. Yet, this relationship was systematically stronger for verbal working memory, as compared to visual working memory, suggesting that a well-developed verbal working memory leads to lexically and grammatically more accurate language production in preschool children.

## 1. Introduction

Language and executive functions develop intensively at preschool age and are taken as important indicators of children’s cognitive development [1]. For example, they are used as indicators of children’s readiness for school [2,3] and predictors of their future academic performance [4,5,6].

There is a convincing body of research suggesting that executive functions predict the acquisition of reading and writing skills in preschool children and in children attending primary school [7,8]. However, data on the relationship between executive functions and oral language skills at preschool age is limited and contradictory [9,10]. The current study addressed this gap in knowledge and examined the relationship between working memory (as a component of executive functions) and oral narrative skills in 5–6-year-old children.

### 1.1. Language Development

Language production covers a number of linguistic skills, though the ability to produce a coherent and logical narration/story can be considered as the main indicator of language competence [11,12,13,14]. As the narrative skill combines a number of linguistic variables, such as lexical, grammatical, syntactical and pragmatic variables, it is a challenge to assess it as a whole. To address this issue, researchers proposed to examine, separately, the macrostructure and the microstructure of a narrative [14,15]. The microstructure of the narrative includes its lexical and grammatical variables, verbal productivity and grammatical complexity [16], whereas the macrostructure of the narrative refers to a hierarchically organized set of propositions [17].

A number of approaches have been proposed to characterize a narrative’s macrostructure [14,15,16,17,18,19,20]. In the current study, we used three of them: (1) Mandler’s view [18] of a narrative structure as a “goal-attempt-outcome”, (2) Ovchinnikova’s framework [19] that assesses the completeness of children’s stories, and (3) Akhutina’s neurolinguistic perspective [20] to analyze the quality of narratives. Ovchinnikova distinguishes a complete, a simplified and a distorted type of the narrative (based on a description of one or a series of pictures) [19]. According to Akhutina, storytelling can be characterized by its semantic completeness, semantic adequacy, story programming, speech pace and its lexical and grammatical accuracy [20]. Semantic adequacy combines two aspects: semantic adequacy A, which is associated with the enlargement of an oral statement (how detailed is a child’s description of an idea); and semantic adequacy B, which is associated with semantic distortions.

To examine narrative skills in children, we chose two tasks traditionally used in neurolinguistics and psycholinguistics, i.e., to create a story based on one or a series of pictures and to retell a story [21]. In line with the above-presented frameworks, we assessed the following variables of narration: narrative type (complete, simplified or distorted) and compliance with the narrative structure (goal-attempt-outcome), semantic completeness, semantic adequacy, story programming (text coherence), story duration (in seconds), speech pace and both lexical and grammatical accuracy (particularly, the number of lexical and grammatical mistakes, paragrammatisms). In order to fully characterize oral language development, we, in addition, assessed children’s verbal fluency (number of words, syntagmas and sentences in the elicited narration).

### 1.2. Working Memory

Working memory is considered to be one of the main components of executive functions that allows to process, store and update information, in real time, in order to accomplish a task [22,23,24]. According to Miyake’s model, executive functions (inhibition, cognitive flexibility, updating and monitoring of working memory) can be defined as a group of cognitive skills that enable problem solving and adaptation in new situations [22]. Within this framework, working memory is defined as one of the target executive functions, as it allows to monitor incoming information based on its relevance for the task, to maintain it, to actively manipulate it and to renew it, if necessary, with new contents [22]. Thus, working memory involves maintenance of the information that is no longer perceptually present in a person’s mind, and its processing or manipulation [23].

There are different models of working memory. For instance, Baddeley identified three components of working memory: phonological buffer, visual-spatial sketchpad and an episodic buffer [24]. Diamond, on the other hand, distinguished two types of working memory—visual and verbal—involved in processing and storage of visual-spatial and verbal information, respectively [23]. Both models have two common components, verbal and visual, which represent two distinct mental processes. The majority of previous studies have examined the relationship between either visual or verbal working memory and language development; in the current research, we aimed to examine both components of working memory capacity in relation to language competence.

### 1.3. Relationship Between Language Development and Working Memory

Previous research has shown a robust relationship between language and executive functions, in general, and between language and working memory, in particular (at least in English-speaking participants) [25,26,27,28,29,30,31]. For example, Alloway and colleagues [27] revealed that children with low working memory scores had lower verbal ability. However, a detailed analysis of the studies revealed varying results across tasks and methods. For instance, while a positive relationship has been consistently reported between executive functions, in general, and comprehension of a verbal statement, in particular, in both children and adults [29,30], research on the relationship between working memory (or her subcomponents) and language narrative skills (comprehension and/or production) is not conclusive. For example, studies focusing on preschoolers and primary-school children reported a relationship between working memory, on the one hand, and vocabulary [31,32], reading skills and verbal intelligence [26,33], on the other hand. 

Yet, the main two components of working memory—the visual-spatial and the verbal [23]—appear to contribute differently to the development of language skills [34]. The study by Adams and Gathercole [34], for example, revealed that, in 4-year-old children, verbal working memory (word span) had numerically stronger correlation with speech production skills (number of different words, syntactic constructions, number of utterances), as compared to visuo-spatial short-term memory (assessed with the Corsi Blocks task), suggesting that verbal working memory might have stronger contribution to language development than visual working memory. A longitudinal study by Cain, Oakhill and Bryant [29] revealed that, in 8–11-year-old children, verbal (sentence-span) working memory had a higher number of significant relationships with language variables, as compared to digital-span working memory, suggesting that verbal working memory might facilitate processing of complex linguistic skills. Note that there was no relationship between digital working memory and component skills or reading comprehension in 7.5-year-old children, suggesting that, in novice readers, only verbal working memory predicts comprehension of a written text. Studies in young children and toddlers have reported a relationship between working memory and spontaneous language production by showing that, during a play, children with high levels of phonological (verbal) working memory used more diverse words, had more complex grammatical structures and used longer sentences than children with low levels of working memory [26]. These studies suggest that verbal working memory might support the development of (complex) linguistic skills related to sentence processing to a larger extent than other types of working memory (e.g., digital-span working memory, visuo-spatial short-term memory).

Although there is a growing body of research on the relationship between working memory and language development, only a few studies thus far have examined the relationship between working memory and children’s ability to produce a spontaneous monologue speech, as, for example, when children compose a story (a narrative) [35], or retell it [36]. Among these studies, the overwhelming majority examined this relationship in atypically developing populations or in a comparative paradigm (e.g., atypically vs. typically developing children) [33,37,38,39,40,41,42]. For example, Dodwell and Bavin [38] reported a significant relationship between various working memory tasks and children’s capacity to retell and to create a story (based on pictures). It is important to notice that among different working-memory tasks, the sentence-repetition task was the best predictor of children’s capacity to retell and understand stories. Other research, however, failed to reveal a relationship between working memory and children’s ability to retell a story [41]. Whitely and Colozzo [43] showed that the ability to update information in working memory (and not the age or short-term memory capacity) was associated with the number of correct references to the characters in a made-up story, in both preschool and in primary-school children. Other research revealed that preschoolers with higher working memory capacity formulated answers (to the questions) better than children with lower working memory capacity [44]. Similarly to the studies in children, research on story retelling in typically developing adolescents revealed a relationship between the volume of working memory and the ability to create complex sentences while composing a picture-based story [35].

In sum, research on the relationship between working memory and story creating in preschoolers is sparse, and the results of existing studies are not conclusive due, mainly, to differences in tasks used to elicit narration (retelling, composing a story based on one or a series of pictures) and in populations (atypically developing children and matched on different factors peers [38,41,42]), which limits the generalizability of the results to typically developing children (without language delays). Finally, in the majority of the above-mentioned studies, the two components (visual and verbal) of working memory were analyzed conjunctly, which does not allow to evaluate their respective contribution to the development of spontaneous language production.

### 1.4. Aims and Hypotheses

The goal of our study was to examine the respective role of visual and verbal working memory capacities in the development of spontaneous language production skills in 5–6-year-old children. First, in line with previous research [34], we expected a relationship between the two components of working memory and language outcomes; however, we expected that the strength of the relationship would fluctuate across linguistic variables and between the two components of working memory, with the verbal working memory revealing a stronger relationship. Second, we hypothesized that there would be a relationship between different linguistic variables of narration across an unprepared (a story based on one or a series of pictures) and an already prepared story (retelling) [21]. We opted for a within-subject design, that is, all children performed in all tasks.

## 2. Materials and Methods

### 2.1. Participants

A total of 287 children were recruited to take part in the study; data from 18 children were excluded from the analyses (see Data analysis for details). The final sample consisted of 269 monolingual typically developing children (i.e., not having delays in language and cognitive development) aged from 5 to 6 years (M = 5.6 years; Sd = 0.48), who attended different kindergartens in Moscow. There were 133 boys and 136 girls.

### 2.2. Measures

#### 2.2.1. Working Memory Assessment

Working memory capacity was assessed using two methods, selected from the subtests of the diagnostic complex NEPSY-II [45]:

(1) The subtest “Sentences Repetition” aimed to assess verbal working memory. This technique uses 17 sentences, gradually increasing in their complexity (sentences become longer and syntactically more complex). For example, while the first sentence consists of 2 words and has a simple structure—“Good night”, the twelfth sentence consists of 14 words and has a complex structure—“The woman, who stands next to a man in a green jacket, is my aunt”. Omitting a word, replacing it or adding another word was considered as an error. Changes in word order, as well as word relocation, were also considered as an error. An accurately reproduced sentence received 2 points, a sentence containing 1 or 2 errors received 1 point, a sentence with 3 errors or more received 0 points. If a child received 0 points for four consecutive sentences, then the test was terminated.
(2) The subtest “Memory for Designs” aimed to assess visual working memory. Two parameters of visual memory were measured—memorization of “pictures” (selection of pictures, as in a presented sample, from an array of similar pictures) and memorization of a spatial arrangement of the pictures (recall the cards’ position in a sample). For each task, 2 points were awarded for each correctly chosen card (called “content”) and 1 for each correctly indicated place (called “spatial”). Two bonus points were given on each trial if a child correctly selected the card and placed it on its right place. As a result, four estimates were obtained for visual working memory: (1) a content score, (2) a spatial score, (3) a bonus score and (4) a total score (sum of all points in all tasks), as described in the NEPSY-II battery.

#### 2.2.2. Language Assessment

As mentioned in the Introduction section, the assessment of the oral monologue was done using Russian neuropsychological methods [46]. These methods are based on a theory of a systematic structure of high mental functions and are aimed to identify the development of their components [20,21].

(1) The “Story Retelling” technique [47] is sensitive to the child’s ability to correctly perceive, retain and recall verbal information and lexical items (auditory processing of information), and it depends on an ability to listen carefully to the story, to highlight the key message (programming and control), and to build a retelling structure and syntactic constructions (serial speech organization). In this task, the fable “The Jackdaw and the Pigeons” by L.N. Tolstoy was used. The following instruction was given to children by the experimenter: “I will read a story now, please listen to it carefully, and then you will have to retell it to me”. In the absence of an answer after the first run, the story was read a second time; there were a maximum of three reading rounds. Note that the number of reading rounds that children required was not related to their working memory capacity (x^2^ = 8.706, *р* = 0.191, see Table S2: Number of story readings for children with different working memory levels).
(2) The method “Creating a story based on one or a series of pictures” [47] consisted of three series of pictures (“The Tower”, “The Cat and the Dog”, “The Broken Cup”), that children had to assemble in their logical sequence of events, and to build a story based on those pictures.

This technique included 3 tasks:

**1.**  In the task “The Cat and the Dog” [48], a series of pictures was laid out in front of a child and he/she had to tell what had happened.
**2.**  In the task “The Tower” [49], a child was given 3 pictures, united by one plot, and he/she had to, first, arrange them in the correct order (what happened at the beginning, what happened afterwards and how it all ended), and then he/she had to tell a story about what happened.
**3.**  In the task “Broken Cup” [49], one picture showing a certain situation was put on the table, in front of a child, then a child had to tell what he believed had happened before.

In each task, after that a child had finished his/her story, the experimenter asked questions to ensure that the child understood the content and could accurately predict further development of the story. It is important to note that if a child determined the sequence of events incorrectly, he/she was not corrected.

The following linguistic variables were analyzed in both linguistics tasks (1) and (2). First, we assessed linguistic variables characterizing a text’s macrostructure: semantic completeness, semantic adequacy (A and B, see Introduction), story programming and speech pace, number of words, paragrammatisms and simple sentences [50], narrative’s type (complete, simplified or distorted [51]) and compliance with the narrative structure [18,50]. Second, we examined text’s microstructure: lexical accuracy (how correctly a child used words) and grammatical accuracy (how many syntactic errors, including missing a verb predicate a child made), number of syntagmas (for more details on scoring see Table S1: Scoring procedure of linguistic variables). Going ahead of the analyses, we would like to mention that high scores for the semantic adequacy, programming, grammatical and lexical accuracy reflected the number of mistakes, so they indicated low performance. 

### 2.3. Procedure

All tasks were performed in the second half of the school year, during three individual meetings with each child (each lasting 20–25 min), in a quiet room of a child’s kindergarten. Children’s stories were recorded on a dictaphone. The order of the tasks was counterbalanced. On each day, children were assessed either on their language production, visual or verbal working memory. The study was approved by the Ethics Committee of the Faculty of Psychology at Lomonosov Moscow State University (the approval No: 2018/42). 

### 2.4. Data Analysis

First, we examined participants’ background data and excluded children according to our general exclusion criteria (being bilingual or having language/cognitive impairments). As a result, four bilingual children were excluded from the sample. Then, experienced testers transcribed children’s stories. A portion of the transcribed texts was randomly selected and reevaluated by an experienced researcher (professor, a specialist in the field of neuropsychology and psycholinguistics). The results revealed a very high consistency between the scores of the testers and the expert. When the scores did not match the corresponding data were discarded from the analyses (14 subjects). Then, for the remaining children, the transcribed tests were assessed against the linguistic variables and average scores were computed for each child and linguistic variable. Therefore, for each of four narratives (one in story retelling and three in story creation), there were 14 linguistic variables per child. For details on how we scored children’s answers see Table S1: Scoring procedure of linguistic variables.

In both verbal and visual working memory tasks, the experimenter filled in a protocol while the child was providing answers. The protocols were examined using the procedures described in the NEPSY-II battery [45], and, then, for each child, we computed one score for verbal working memory and four scores for visual working memory (see Section 2.2.1 for details). 

## 3. Results

### 3.1. Correlation Analysis of Working Memory and Oral Language Skills in Preschoolers

At the first stage of the analysis, we checked the consistency between the four stories (one from retelling and three from creating a story on a series of pictures) for the 14 linguistic variables. All of them showed consistency by the Alpha-Cronbach criterion above 0.50; then, we computed, for each linguistic variable, one cumulative score across the four narratives.

At the second step, we performed a correlation analysis between the cumulative scores of oral narrative language development and the scores obtained in the working memory tasks: the total score for the visual and the total score for the verbal working memory (Table 1).

As can be seen in Table 1, both types of working memory (visual and verbal) were related to semantic completeness, semantic adequacy, story programming, number of words, sentences and syntagmas, as well as to the compliance with the narrative structure and the type of narrative. These relationships show that the most common and universal indicators of a narrative’s macrostructure are associated with the general (both visual and verbal) working memory capacity. As for the lexical and grammatical accuracy, as well as for the semantic adequacy B, they primarily correlated with verbal working memory.

To examine the strength of the relationship between each type of working memory and the linguistic variables, we separated the linguistic variables into two groups of variables: those that characterize text’s macrostructure and those that characterize text’s microstructure (as in the Methods), and computed an averaged correlation coefficient for the verbal and visual working memory (see Table 1). Then we compared the coefficients between the two types of working memory using the online platform [52]. The results revealed that, when compared to visual working memory, verbal working memory had a stronger relationship with the linguistic variables of the text’s macrostructure, (r = 0.34 vs r = 0.16, *p* = 0.0013) and with the linguistic variables of the text’s microstructure (r = 0.12 vs r = 0.28, *p* = 0.0027). These results suggest that, in pre-school children, verbal working memory is associated with oral narrative skills more strongly than visual working memory.

### 3.2. Differences in the Development of Oral Language in Preschoolers with Different Levels of Working Memory

Cluster analysis (the K-means method was used) of the results in the working memory tasks was also carried out (using the K-means method). It allowed us to identify 3 groups of children with low, medium and high levels of working memory (Table 2).

It is important to mention that pair-wise comparison of clusters (the Mann-Whitney test) showed no significant difference in the level of verbal working memory in children with moderate and high levels of working memory. The rest of the indicators between all clusters were significantly different. Table 3 summarizes data on the significant differences in the indicators of oral language competence in preschoolers with different levels of working memory capacity.

The pair-wise analysis of the clusters did not reveal statistically significant differences between children with medium and high working memory capacities, which can be explained by the lack of differences in the levels of verbal working memory between groups. However, children with low levels of working memory differed significantly from those who had medium and high levels of working memory due to differences in a variety of linguistic variables: semantic completeness and adequacy, story programming, number of words and sentences, adherence to the structure of the narrative and the type of narrative. At the same time, children with different levels of working memory did not differ in terms of speech pace and narrative time, semantic adequacy (B and general) and grammatical and lexical design of the story.

In sum, similar results were obtained both in correlation and in cluster analysis revealing that some variables of children’s oral language development, as, semantic completeness of the narrative, its adequacy, programming, word count and number of sentences in the story, narrative structure and type correlated with both types of working memory capacity in 5–6-year-old children.

## 4. Discussion

This study aimed to examine the relationship between oral narrative language skills and working memory capacity in 5–6-year-old children. We hypothesized, first, that language outcomes would be related to working memory capacity (both visual and verbal), although the relationship would not be systematic across different linguistic variables. Second, we expected more numerous and stronger relationships between linguistic variables and verbal working memory, as compared to visual working memory. Finally, we hypothesized that the linguistic variables measured in both unprepared (creating a story based on one or a series of pictures) and prepared (story retelling) linguistic materials would be related with each other across subjects.

Importantly, our results revealed that both—visual and verbal—working memory capacities were related to a number of linguistic variables. In particular, they were related to semantic completeness and semantic adequacy of narration, to programming, number of words, sentences and syntagmas, as well as to the adherence to the narrative structure and to the type of narrative. These results support Adams and Gathercole’s claim [36] that memory resources associated with language development cannot be completely aligned with verbal working memory and they need to rely on visual working memory too. According to Luria’s theory [48] of a systemic structure of higher mental functions, language, as a higher mental function, should be subserved by both visual and verbal working memory. In addition, creating a story based on a series of pictures involves visual-spatial analysis of picture details, which might explain the involvement of visual working memory capacity and its relationship to children’s performance in the linguistic task. Story retelling and story creation (from pictures), on the other hand, both involve sentence planning, that comprises maintenance and on-line manipulation of relatively complex syntactic structures. Maintenance of a syntactic structure was a key component to successful performance in the verbal working memory task, as participants had to repeat sentences increasing in their syntactic complexity and high scores were attributed to those who were able to repeat sentences accurately, with no word omissions.

Our results indicate that the most common and universal indicators of a narrative’s macrostructure are associated more strongly to verbal than to visual working memory capacity. In general, these results are in line with the results of previous studies conducted in children and adolescents [40,43,44], and extend them to previously unexamined narrative variables, such as the type and the completeness of a narrative, its semantic completeness, semantic adequacy and story programming. These results are new and should be confirmed and replicated in future research.

The results of the correlation analysis confirmed our expectations: verbal working memory had more numerous and stronger relationships with the linguistic variables than visual working memory. Note that some linguistic variables, as, for example, lexical and grammatical story design, as well as the semantic adequacy B, correlated with verbal working memory only; they were not associated with visual working memory. This result shows that visual working memory might not be associated with the development of ideas about the grammatical structure of sentences in preschoolers. However, understanding the concepts of grammar and being able to use grammar to design one’s own sentence may represent two overlapping yet different competences, with the former revealing more metalinguistic skills.

In our results, verbal working memory was associated with the semantic adequacy B, unlike visual working memory. The penalties for the semantic adequacy A parameter were applied when a child skipped the key semantic elements or when the semantic breadth was insufficient. For the semantic adequacy B, penalties were applied for an unrealistic interpretation of the picture or for breaking the connection between the events. Our results revealed that visual working memory was associated primarily with the semantic breadth of the utterances and not with the understanding of pictures’ meaning. Verbal working memory, on the other hand, was related to the understanding of the story depicted in pictures. This result may be explained by differences in the methods: in the verbal working memory task, a child can/has to rely on a sentence’s meaning, whereas in the visual working memory task, a child has to memorize pictures of abstract objects rather than real objects.

The question about cultural and language family specificity of our data remains open. In the current study, the relationship between working memory and oral language production was assessed in Russian and whether the results can be generalized to other languages remains to be addressed in future research. For instance, some studies suggest that narratives in children are language and culture specific [53], while other studies showed no language differences (e.g., English and Swedish in reference [54]). Future research needs to examine this issue by collecting and comparing data on the relationship between working memory and oral language skills in other European and non-European languages.

## 5. Conclusions

To conclude, the current study revealed that both visual and verbal working memory components were related to a number of oral language outcomes, with verbal working memory having more numerous and stronger relationships. In particular, verbal working memory was strongly related to lexical and grammatical accuracy in oral (narrative) language skills in 5–6-year-old children, suggesting that a well-developed verbal working memory leads to lexically and grammatically accurate spontaneous language production.

## Figures and Tables

**Table 1:** Correlation of oral linguistic variables and working memory in preschool children (Pearson criterion).

Linguistic Variables	Visual Working Memory	Verbal Working Memory
semantic completeness	0.23 **	0.50 **
semantic adequacy А ***	−0.18 **	−0.41 **
semantic adequacy B ***	−0.11	−0.23 **
general semantic adequacy ***	−0.16 *	−0.41 **
Programming ***	−0.18 **	−0.45 **
story time	0.06	0.06
speech pace	0.15 *	0.27 **
number of words	0.16 **	0.25 **
number of simple sentences	0.15 *	0.20 **
narrative structure	0.26 **	0.43 **
narrative type	0.25**	0.49 **
**Macrostructure (average)**	0.16	0.34
grammatical accuracy ***	−0.12 *	−0.31 **
number of syntagmas	0.15 *	0.22 **
lexical accuracy ***	−0.08	−0.32 **
**Microstructure (average)**	0.12	0.28

* *p* < 0.05 (2-tailed); ** *p* < 0.01 (2-tailed), statistically significant correlations **in bold**., *** Please note that high scores indicate low performance.

**Table 2:** Final centers of clusters based on the success of the children performing the working memory (WM) tests.

	Low WM Level	Medium WM Level	High WM Level
Visual WM Content	33.52	37.61	44.75
Visual WM Spatial	14.85	19.98	23.03
Visual WM Bonus	6.50	18.06	37.09
Visual WM Total Score	54.87	75.65	104.88
Verbal WM	17	19	20
Number of children	92	113	64

**Table 3:** Significant differences in linguistic variables in preschoolers with different levels of general working memory capacity.

Linguistic Variables	Low WM Level		Medium WM Level		High WM Level	
	M	SD	M	SD	M	SD
semantic completeness	52.43	21.47	59.69	17.10	60.51	17.11
semantic adequacy А *	5.26	2.01	4.72	1.61	4.56	1.60
story programming *	7.90	2.27	7.34	1.94	7.10	2.00
number of words	68.37	32.39	76.09	31.26	77.54	27.54
number of sentences	13.65	5.62	14.89	5.05	14.92	4.38
narrative structure	1.72	1.50	2.33	1.55	2.49	1.41
narrative type	1.74	1.47	2.37	1.47	2.51	1.58

* Please note that high scores indicate low performance.

## Supplementary Materials

The following are available online at https://www.mdpi.com/2076-328X/10/2/52/s1, Table S1: Scoring procedure of linguistic variables, Table S2: Number of story readings for children with different working memory (WM) levels.
