# Supplementary material for: The Relationship between Executive Functions and Language Production in 5–6-Year-Old Children: Insights from Working Memory and Storytelling
Source: Behav Sci (Basel). 2020 Feb 5;10(2):52. doi: 10.3390/bs10020052 (PMC7071471; doi:10.3390/bs10020052)
Supplement: Supplementary file 1 [file behavsci-10-00052-s001.pdf]

Table S1. Scoring procedure of linguistic variables.

Linguistic variables	Description
semantic completeness (0 – 30 points)	in each text there are 10 essential semantic elements that children are supposed to indicate. If a child names this element by him/herself s/he is given 3 points, if s/he mentions it after testor's question s/he is given 1,5 points, if s/he does not mention it at all s/he is given 0 points
semantic adequacy A (0 – 3 points)	0 – a child does not commit any semantic mistakes, his/her story reflects all the necessary semantic units;<br>1 point – a child commits 1 or 2 mistakes, his/her story is complete, still 1-2 semantic units are missing;<br>2 points – 2-3 rude mistakes, some important information is missing;<br>3 points – event description does not correspond to the images;
semantic adequacy B (0 – 3 points)	0 – a child does not commit any semantic mistakes;<br>1 point – 1 mistake or 1 case of semantic distortion;<br>2 points – 2-3 mistakes or 2-3 cases of semantic distortion;<br>3 points – unrealistical event description;
programming (0 – 3 points)	0 – a child produces a whole coherent story;<br>1 point – a child misses some semantic or logical link;<br>2 points – misses important semantic elements, makes semantic repetitions;<br>3 points – gives no coherent story
story time	in seconds
speech pace	verbal productivity/ story duration ratio
number of words (verbal productivity)	total number of words
number of simple sentences	a simple sentence has been defined as a sentence that contains one subject and one predicate;
narrative structure (0-2 points)	It means a compliance with the narrative structure (goal-attempt-outcome).<br>0 point – zero or one narrative structural elements;<br>1 point – two narrative structure elements;<br>2 point – all the narrative structure elements;
narrative type (0-2 points)	0 point - distorted narrative;<br>1 point – simplified narrative;

	2 point – complete narrative;
grammatical accuracy (0 – 3 points)	0 point – a child produces a grammatically correct story with various grammatical structures;<br>1 point – a child produces a grammatically correct story, but the sentences are very short and there is no grammatical diversity;<br>2 points – a child makes some grammatical mistakes, simplifies the grammatical structure;<br>3 points – a child makes many grammatical mistakes, omits predicates;
number of syntagmas	number of verbs and their amplifications
lexical accuracy (0 – 3 points)	0 point – a child produces a lexically correct story with various and correct words;<br>1 point – a child searches the correct words or makes some lexical paraphrases;<br>2 points – a child searches the correct words and makes lexical paraphrases;<br>3 points – many lexical mistakes and poor vocabulary;

Table S2. Number of story readings for children with different working memory (WM) levels.

Number of story readings	Low WM level	Medium WM level	High WM level
1	45.7%	56.6%	53.1%
2	25.0%	23.0%	26.6%
3	27.2%	20.4%	20.3%
